# Influences of Extrusion and Silver Content on the Degradation of Mg-Ag Alloys *In Vitro* and *In Vivo*

**DOI:** 10.1155/2022/2557518

**Published:** 2022-04-23

**Authors:** Guanqi Liu, Jianmin Han, Xiaodong Yu, Shenpo Yuan, Zhihua Nie, Tiancheng Qiu, Ziyu Yan, Chengwen Tan, Chuanbin Guo

**Affiliations:** ^1^School of Materials Science and Engineering, Beijing Institute of Technology, Beijing 100081, China; ^2^Department of Dental Materials, Peking University School and Hospital of Stomatology, National Center of Stomatology, National Clinical Research Center for Oral Diseases, National Engineering Laboratory for Digital and Material Technology of Stomatology, Beijing Key Laboratory of Digital Stomatology, Research Center of Engineering and Technology for Computerized Dentistry Ministry of Health, NMPA Key Laboratory for Dental Materials, Beijing 100081, China; ^3^Department of Oral and Maxillofacial Surgery, Peking University School and Hospital of Stomatology, National Center of Stomatology, National Clinical Research Center for Oral Diseases, National Engineering Laboratory for Digital and Material Technology of Stomatology, Beijing Key Laboratory of Digital Stomatology, Research Center of Engineering and Technology for Computerized Dentistry Ministry of Health, NMPA Key Laboratory for Dental Materials, Beijing 100081, China

## Abstract

Binary magnesium-silver (Mg-Ag) alloys were designed as antibacterial materials for biomedical implant applications. In the present study, we focused on the effects of extrusion (extrusion ratio (ER): 1, 7.1, and 72.2) and Ag content (Ag = 0, 3, and 6 wt.%) on the degradation of Mg-Ag alloys *in vitro* and *in vivo* via microstructure characterization and corrosion/degradation measurements. The results showed that the Ag promoted a galvanic reaction with the Mg matrix to accelerate degradation or formed a protective oxide mesh texture to inhibit degradation, especially *in vivo*. Ag might also be beneficial for product crystallization, biomineralization, and organic matter deposition. For pure Mg, extrusion produced a more refined grain and decreased the degradation rate. For the Mg-Ag alloys, a low extrusion ratio (7.1) accelerated the degradation caused by the increase in the proportion of the precipitate. This promoted the release of Mg^2+^ and Ag^+^, which led to more deposition of organic matter and calcium phosphate, but also more H_2_ bubbles, which led to disturbance of product deposition in some local positions or even inflammatory reactions. Extrusion at a higher ratio (72.2) dissolved the precipitates. This resulted in moderate degradation rates and less gas production, which promoted osteogenesis without an obvious inflammation reaction.

## 1. Introduction

Magnesium (Mg) alloys have aroused much interest as biodegradation materials for use in bone and cardiovascular applications [1–4]. Their degradability means that a secondary surgery for implant removal can be avoided. Furthermore, Mg-alloys exhibit similar densities and elastic moduli to cortical bone, which could reduce the “stress shielding” effect [5, 6]. They also have good biocompatibility and promote osteogenesis [7–9]. However, their rapid degradation rate, especially at the initial stage, accompanied by the large release of hydrogen gas, is still the main concern for their applications [10]. Their degradation would reduce their mechanical integrity, leading to premature implant fracture [11, 12], and implant-associated orthopedic surgery infections [13]. Thus, Mg-alloys with a low early degradation rate and good antibacterial properties are urgently required.

Silver (Ag) is considered a good antimicrobial element and has been widely used as a treatment for burns, open wounds, and chronic ulcer infections for centuries [14, 15]. Silver ions and silver nanoparticles (AgNPs) influence the membrane and proteins of bacteria, interfere with their DNA expression, and inhibit their respiratory processes [16–18].

Ag addition to Mg could also improve the mechanical properties and regulate the corrosion rates when a suitable preparation process is conducted. Wiese et al. [19] showed that Ag could refine the grain and decrease the lattice parameters of Mg-Ag alloys, as well as the c/a ratio of the hcp lattice structure, which led to an increase in yield strength and elongation, especially in the tensile strength test along the extrusion direction. Estrin et al. [20] showed that Mg-2Ag and Mg-4Ag, extruded at a speed of 2.2 mm/s, had a low corrosion rate of about 1.0 mm/year over 7 days of immersion in the biological environment. However, more details of the microstructures and degradation behaviors of Mg-Ag alloys *in vitro* and *in vivo* should be acquired. For example, how the Ag content influences the corrosion products and rates remains unclear. Therefore, to increase the mechanical properties of Mg-Ag alloys, some deformation processes, such as extrusion, have been applied during alloy preparation [21, 22]. These plastic deformation technologies could effectively refine the grains and modify the precipitates in the alloys, thereby representing factors that might influence the corrosion behavior of the alloys, which requires further study.

Previous studies also reported that Mg-Ag alloys have negligible cytotoxicity, good cytocompatibility, and antibacterial activity, and show potential as *in vivo* implants [23, 24]. Tie et al. [23] showed that the Mg-Ag alloys had better antibacterial properties with increasing Ag content, and that T4 (solution treatment) treated Mg-2Ag and Mg-4Ag may be potential antibacterial biodegradable materials because of their good comprehensive properties. Jähn et al. [24] reported that Mg-2Ag intramedullary nails were suitable implants for femoral fracture fixation in mice, although augmented callus formation occurred during healing. However, the histological response after implantation with different Ag contents and deformation ratios for Mg-Ag alloys *in vivo* is seldom studied and thus requires systematic research.

Therefore, in the present study, a series of Mg-Ag binary alloys with different Ag contents (Ag = 0, 3, and 6 wt.%) and extrusion ratios (1, 7.1, and 72.2) was prepared and tested. Dulbecco's modified Eagle's medium (DMEM) + 10% fetal bovine serum (FBS) at 37°C and 5% CO_2_ was chosen to simulate the physical environment, which was regarded as a reliable electrolyte for *in vitro* corrosion tests [25, 26]. A rat femoral condyle model was used for *in vivo* evaluation because of its ease of operation and the good fixation of material after implantation, which facilitates characterization of the implants. The results showed that the Ag content and extrusion ratio significantly regulated the alloy microstructure (such as precipitate level and grain size), thereby affecting its degradation behavior and histological properties. The differences of Mg-Ag alloy degradation *in vivo* and *in vitro* were also compared. This study provides further guidance for the use of Mg-Ag alloys as orthopedic implant materials.

## 2. Materials and Methods

### 2.1. Material Production and Characterization

Mg-xAg alloys (*x* = 0, 3, and 6 wt.%) were prepared by permanent mold gravity casting. Subsequently, the ingots were homogenized in a resistance furnace at 430°C for 16 hours, followed by water quenching (referred to as T4 treatment). The alloy ingots were also extruded at 300°C from Φ85 mm (as cast) to Φ32 mm (extrusion ratio (ER): 7.1) and Φ10 mm (extrusion ratio (ER): 72.2), at a speed of 5–7 mm/s.

The chemical composition was tested using an inductively coupled plasma-atomic emission spectrometer (ICP-AES, PerkinElmer, Optima 7300DV, Houston, TX, USA). The average densities of the Mg alloy samples were determined in ethanol using the Archimedean principle. The results are shown in Table 1.

The specimens were mounted, ground with SiC paper up to 5000 grit, polished using a diamond paste down to 1.5 *μ*m, and then lightly etched in a picric acid solution (1 g of picric acid, 1.3 g of acetic acid, 7 mL of H_2_O, and 20 mL of ethanol). A field emission scanning electron microscope (SEM, QUANTA 200F, FEI, Hillsboro, OR, USA) was used to observe the microstructure of the specimens. Image J software (public domain; National Institutes of Health, Bethesda, MD, USA) was used to calculate the proportion of precipitates in the alloys from scanning electron microscopy (SEM) images. Five images per sample were measured and averaged. Then, the specimens were polished using argon ions in a Precision Ion Polishing System 691 (Gatan, Pleasanton, CA, USA). A JEOL JSM-7001F scanning electron microscope with a TSL OIM 6.2 system for electron backscatter diffraction (EBSD; Jeol, Akishima, Japan) was used to analyze the grain sizes of alloys. The X-ray diffraction (XRD) spectra were tested via a Bruker D8 Advance X-ray diffractometer (Bruker) to identify the phase of the samples. The measurements were carried out with Cu K*α* radiation in the range of 2*θ* from 10° to 90° (incidence angle 3°, step 0.02°, exposure time 1 s). Thereafter, the ingots were machined along the extrusion direction to Φ10 mm × 5 mm for the *in vitro* corrosion test, and to Φ2 mm × 5 mm for the *in vivo* corrosion test.

### 2.2. Electrochemical Experiments

The test was conducted in DMEM (Sigma-Aldrich Chemie, Taufkirchen, Germany) and GlutaMAX with 10% (by volume) FBS (Zhejiang Tianhang Biotechnology Co. Ltd., Zhejiang, China) under cell culture conditions (37 ± 0.5°C, 5% CO_2_, 95% relative humidity). The volume of each corrosion solution was 200 mL.

The electrochemical test was performed using an electrochemistry workstation (PARSTAT 2273, Princeton, NJ, USA). The samples (Φ10 mm × 5 mm) were molded into epoxy resin with only one side of 0.785 cm^2^ exposed for the test. The exposed sides were ground with 2000 grit, and all the samples were disinfected using 75% ethanol for 20 min. A traditional three-electrode system was established with the sample as the working electrode, a saturated calomel electrode (SCE) as the reference electrode, and a 10 mm × 10 mm Pt foil as the counter electrode, respectively. The open circuit potential (OCP) was first measured until the potential was stable. Then, the potentiodynamic polarization curve was tested using a scanning rate of 0.5 mV/s and a scanning range of −0.25 V∼−1.25 V relative to the OCP. The electrochemical tests were performed 1 day after immersion, and the data were analyzed using Origin v.8.0 (MicroCal, Northampton, MA, USA).

### 2.3. Immersion Experiment

For the immersion test, samples with the same size (Φ10 mm × 5 mm) were cut from the bars, ground with 2000 grit SiC paper for each surface, and sterilized using 75% ethanol for 20 min. The immersion conditions were the same as those in section 2.2. The V/S ratio (solution volume/sample surface area) of these samples was 63.66 mL/cm^2^. The samples were immersed for 1, 4, 8, and 16 days. Five samples were set for each time point. The solution was changed every 4 days to provide a semistatic immersion test and to avoid saturation effects. After immersion, the formed corrosion products were removed by immersion in a chromic acid solution (20 g chromium (VI) oxide, 1 g AgNO_3_, and 100 mL of distilled H_2_O) for 20 min at room temperature. The samples before and after the test were weighed using a precision electronic balance. The corrosion rate (CR) was calculated in mm/year using the equation:

(1) $$\begin{array}{c} CR=\frac{87600\cdot \Delta W}{A\cdot t\cdot \rho }, \end{array}$$

where Δ*W* (g) is the weight change between before immersion and after chromic acid cleaning, *A* is the sample surface area (cm^2^), *t* is the immersion time (h), and *ρ* is the sample density (g/cm^3^).

### 2.4. Corrosion Layer Characterization

After 16 days of immersion, the samples were taken out and dried completely in a vacuum oven. The overall morphology of the samples was characterized using a Canon PowerShot A650 IS camera (Canon Germany GmbH, Krefeld, Germany) and a 3D digital microscope (DVM6A, Leica, Wetzlar, Germany). The surface microstructure and composition of the degradation layer were characterized using SEM (QUANTA 200F, FEI, Hillsboro, OR, USA) and energy-dispersive X-ray spectroscopy (EDS, EDAX, Tilburg, the Netherlands). The cross-section morphology and element mapping were performed via SEM (HITACHI SU8220, Tokyo, Japan) and a Bruker FlatQuad X-ray spectrometer (Bruker, Karlsruhe, Germany).

The phase formations on the sample surfaces were tested via a Bruker D8 Advance X-ray diffractometer (Bruker) to identify the X-ray diffraction (XRD) spectra. The measurements were carried out with Cu K*α* radiation in the range of 2*θ* from 10° to 90° (incidence angle 3°, step 0.02°, exposure time 1 s).

In addition, the surface chemical states of the samples after immersion were characterized by X-ray photoelectron spectroscopy (XPS, PHI QUANTERA-II SXM, ULVAC-PHI, Kanagawa, Japan). The XPS spectra were recorded using Al K*α* radiation (1486.6 eV) as the excitation source. The binding energy of the C 1s signal was used to correct the spectra for charging. The background was subtracted using the Shirley method in all spectra. For each immersion parameter, a survey spectrum and high-resolution spectra for C 1s, Mg 2p, Ca 2p, and Ag 3d signals were measured. The corresponding narrow scans were fitted using Gaussian multipeak fitting in the MultiPak Software (Physical Electronics, Chanhassen, MN, USA).

### 2.5. In Vivo Test

#### 2.5.1. Surgical Procedures

The animal experiments were performed following principles and processes approved by the Animal Care and Use Committee of Peking University. Sprague–Dawley rats were chosen as the standard experimental animals for the *in vivo* tests. Healthy male Sprague–Dawley rats at 8 weeks old (200–220 g) were randomly divided into five groups: the pure Mg group, the Mg-3Ag-as cast group, the Mg-3Ag-ER7.1 group, the Mg-3Ag-ER72.2 group, and the Mg-6Ag-ER7.1 group. Each group comprised 15 rats, which were separated equally for three time-points: 1, 3, and 6 months. The animals were anesthetized by intraperitoneal injection of pentobarbital sodium (50 mg/kg body weight). The rat femoral condyle model was used in this study. Hair on the femoral condyle was removed by shaving, and the exposed area was wiped by swabbing with 3 vol.% povidone-iodine followed by 75 vol.% ethanol for disinfection. We incised the skin, subcutaneous tissue, and periosteum, and exposed the bone via blunt dissection. A trephine burr on a low-speed dental handpiece (Surgic XT, Nakanishi, Japan) with sterile NaCl irrigation was used to create a defect of 2 mm in diameter and 5 mm in depth on each side of the femoral condyle. Then, the Mg-Ag alloys with a size of Φ2 mm × 5 mm were implanted into the defect positions. Finally, the tissue was sutured layer by layer. After surgery, the rats were housed in ventilated rooms and given access to water and food. After 1, 3, and 6 months, the rats were euthanized, and their femurs were harvested and fixed in 10% neutral formalin buffer for microcomputed tomography (CT) and histological analysis.

#### 2.5.2. Micro-CT Analysis

We used an Inveon MM micro-CT system (Siemens, Munich, Germany) to evaluate the implant in femurs. Images were acquired at an effective pixel size of 8.82 *μ*m, 80 kV, 500 *μ*A, and an exposure time of 1500 ms in each of the 360° rotational steps. Based on the transverse two-dimensional projection of the collected samples, three-dimensional reconstruction was carried out using Inveon Research Workplace software (Siemens, Knoxville, TN, USA). For analysis, the center of the cross-section of the implant was defined as the reorientation center of the region of interest. The volumes of remaining materials and the mineralized tissue volume ratios (BV/TVs), which were chosen as the indices of new bone formation, were calculated. The degradation rate (DR) was calculated in mm/year according to the following equation:

(2) $$\begin{array}{c} DR=\frac{\Delta V}{A\cdot t}, \end{array}$$

where Δ*V* (mm^3^) is the implant volume change between before implantation and at the designed implantation time interval. *A* is the initial surface area of the implant (mm^2^), and *t* is the implantation time (year).

#### 2.5.3. Histological Processing

After fixation, the bone samples were divided into two groups. One group was decalcified using 0.5 M EDTA for 15–20 days, then dehydrated in gradient alcohol (50, 60, 70, 80, 90, and 99 vol.%), and embedded in paraffin. The sections were cut (perpendicular to the long axis of the implants) at a 5 *μ*m thickness and subjected to hematoxylin-eosin (H&E) staining. The other group was not decalcified. After dehydration in gradient alcohol, the samples were embedded in autopolymerizing methyl methacrylate (MMA) resin (Wako Pure Chemical Industries, Ltd., Tokyo, Japan). The specimens were cut (perpendicular to the long axis of the implants) into 300 *μ*m thick sections using a diamond saw (STX-202A, Shenyang Kejing Auto-Instrument Co., Ltd., Shenyang, China), adhered to resin slides, ground and polished to a thickness of about 50 *μ*m. The ground specimens were stained with Goldner's trichrome and methylene blue-acid fuchsin. Images were acquired under a microscope (Leica Q500MC, Leica Cambridge Ltd, Cambridge, UK). The histological evaluation mainly focused on the degradation of alloy materials, new bone formation, indicators of inflammation, and the response of surrounding tissues to the implant.

### 2.6. Statistical Analysis

Statistical analysis was conducted using SPSS 18.0 (IBM Corp., Armonk, NY, USA) and differences between groups were analyzed using a one-way analysis of variance (ANOVA). The data are expressed as the mean ± standard deviation (SD).

## 3. Results

### 3.1. Microstructure of the Samples

**Figure 1:**
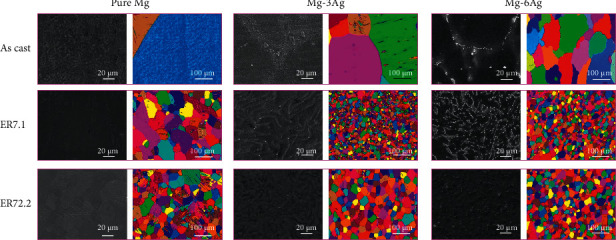
Microstructure of pure Mg, Mg-3Ag, and Mg-6Ag with different extrusion ratios: 1 (as cast), 7.1, and 72.2.

Figure 1 (the left picture of each alloy sample) shows the morphologies of the samples under SEM. Secondary dendrites were observed in the grains of the as cast Ag-containing Mg alloys. The white particles were identified as precipitates. The average proportions of precipitates in the samples are presented in Table 2. The pure Mg did not have precipitates, thus the value was not calculated. For the Mg-Ag alloys, when the ER was 7.1, the average proportion of precipitates increased because of the precipitation process; when the ER increased to 72.2, the average proportion of precipitates decreased because some of the precipitates dissolved in the matrix. In addition, the precipitate proportion in Mg-6Ag was higher than that in Mg-3Ag. Figure 1 (the right picture of each alloy sample) shows the crystalline grain of the samples as assessed using EBSD. The average grain size of each alloy is shown in Table 2. The average grain size of as cast pure Mg was the largest (about 489.99 *μ*m), and the addition of Ag and extrusion significantly refined the grains. A large ER (72.2) might not further refine the grains of the Mg-Ag alloys because recrystallization promoted the dissolution of the Ag, which promoted grain growth.

**Figure 2:**
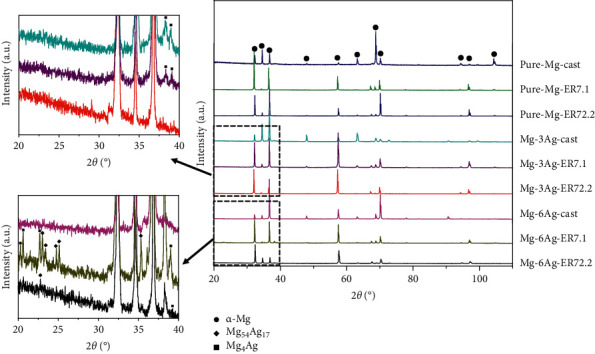
XRD results of pure Mg, Mg-3Ag, and Mg-6Ag with different extrusion ratios: 1 (as cast), 7.1, and 72.2.

Figure 2 shows the XRD analysis results for the nine materials. Only the *α*-Mg structure existed in pure Mg. In the Mg-3Ag and Mg-6Ag alloys, in addition to the matrix *α*-Mg, there were two precipitates: Mg_4_Ag and Mg_54_Ag_17_. However, the content of precipitated Mg_54_Ag_17_ in Mg-3Ag alloys might be very low because the peak was very weak.

### 3.2. In Vitro Degradation Rate as Assessed Using Electrochemical/Weight Loss Measurement

**Figure 3:**
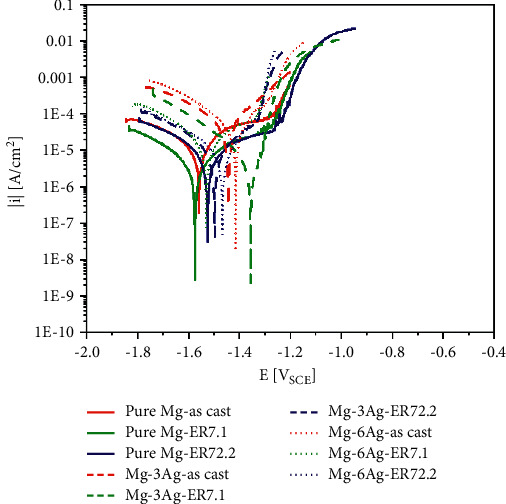
Tafel curves for pure Mg and Mg-Ag alloys after immersion in DMEM + 10% FBS for 1 day.

Figure 3 presents the Tafel curves for the different samples after 1-day of immersion in DMEM + 10% FBS in a cell culture environment. The *E*_corr_ and *I*_corr_ were analyzed via curve fitting in Origin v.8.0, and the results are shown in Table 3.

The Ag increased both the *E*_corr_ and the *I*_corr_. According to Gusieva [27], Ag could enhance the cathodic kinetics, making the intersection of the anodic and cathodic curves occur at a higher voltage and current density. Ag could also form precipitates, resulting in galvanic corrosion with the matrix and accelerated corrosion. Extrusion might alter the grain size and the precipitate proportion for the Mg-Ag alloys, which both influenced the *I*_corr_. The comprehensive effect of extrusion was to reduce *I*_corr_ at this stage.

**Figure 4:**
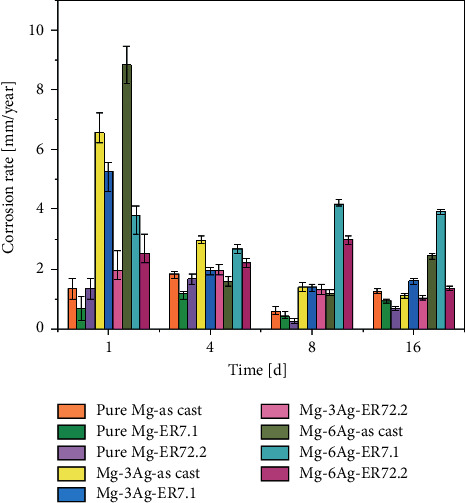
Corrosion rate for pure Mg and Mg-Ag alloys immersion in DMEM + 10% FBS for 1, 4, 8, and 16 days (by the mass loss method).

The corrosion rate was then calculated by mass loss and the results are presented in Figure 4. The corrosion rate changed over time, and the Ag addition and extrusion made the process more complex. Generally, at 1 day after immersion, the corrosion rate of the Mg-Ag alloys was higher than that of pure Mg because of galvanic corrosion between the Mg matrix and the precipitates. As the extrusion ratio increased, the corrosion decreased.

During the 1–16 days, the corrosion rate fluctuated. This was because as corrosion progressed, a corrosion product layer formed on the substrate surface, which slowed down the corrosion rate. The corrosive ions, such as Cl^−^, and the gas bubbles generated by Mg corrosion might destroy the substrate, which increases the corrosion rate. These corrosion behaviors resulted in a fluctuating corrosion rate [28]. At this stage, as the Ag content increased, the corrosion rate also increased. For pure Mg, extrusion refined the grain, which decreased the corrosion rate. However, for the Mg-Ag alloys, extrusion at ER7.1 resulted in a higher corrosion rate than the other two ERs. This was because the increase in precipitates led to more intense galvanic corrosion within the Mg matrix.

### 3.3. Characterization of the Degradation Layer *In Vitro*

**Figure 5:**
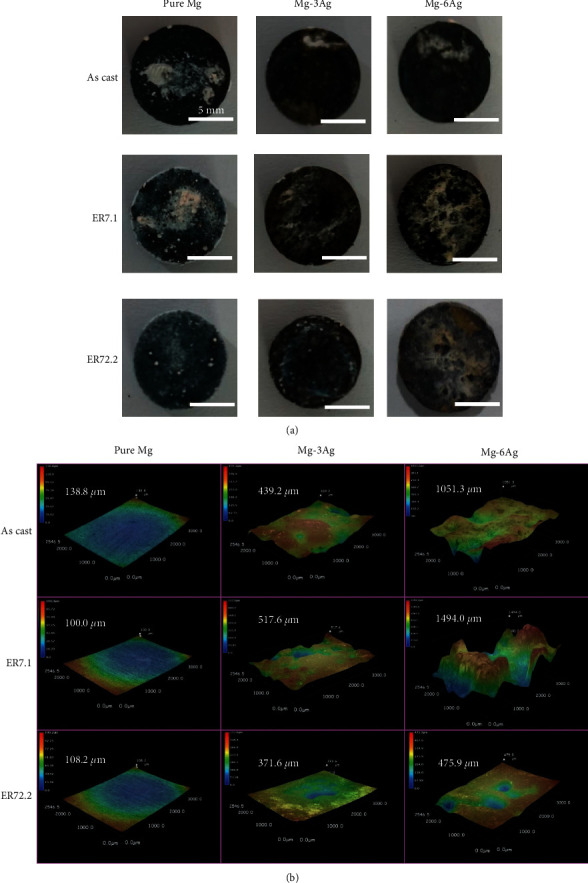
(a) Surface morphology and (b) 3D depth images of the samples after an immersion test for 16 days.

The macromorphologies and the 3D depth images of the samples after immersion are displayed in Figures 5(a) and 5(b). The corrosion product appeared white under natural light. In the pure Mg group, the whole area of the sample was corroded and a uniform and smooth corrosion morphology was observed. The addition of Ag resulted in local corrosion and higher sample surface roughness. The as cast alloys' sample surface roughness was low. An ER of 7.1 resulted in the highest level of surface roughness. This was because the precipitates were coarse and unevenly distributed, leading to serious galvanic corrosion. Further extrusion reduced the surface roughness, mainly because the precipitates were refined and evenly distributed among the Mg matrix.

**Figure 6:**
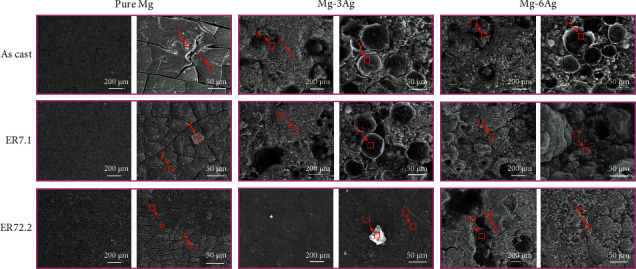
Microstructure observed by SEM of the sample surfaces after an immersion test for 16 days.

Figure 6 shows SEM images of sample surfaces after the immersion test. For the pure Mg group, some corrosion cracks appeared, and the crack size became smaller as the extrusion ratio increased. For the Mg-3Ag group, the as cast and ER7.1 alloys suffered serious local corrosion, and pits left by a large number of H_2_ bubbles were also observed (position 9 and 11), whereas, for the ER72.2 alloy, corrosion was obviously uniform with nearly no pits. The Mg-6Ag group showed more serious corrosion than the Mg-3Ag group, and the ER72.2 alloy also presented more uniform corrosion than the as cast and ER7.1 alloy.

EDS was conducted to characterize the composition of the sample surfaces, and the results are shown in Table 4. Each sample was detected at 2–3 positions and was marked in Figure 6. Besides the Mg and Ag elements, C, O, Na, P, Cl, and Ca were also detected in almost all the samples, while N, Si, S, and K were detected in some individual samples.

Oxygen was identified as the main element on the surface, which resulted from the rapid oxidation of the Mg matrix and the Ag-containing precipitates in the immersion test. Carbon was detected as the second element after O in our sample surfaces. The existence of C implied the deposition or absorption of organic matter. The deposition of Ca and P indicated the formation of calcium phosphates on the alloy surface, which plays an important role in biomineralization [26, 29–31]. The Ca/P ratio was calculated for each position to estimate the calcium phosphate formation process. The main composition in human natural bone is hydroxyapatite with a Ca/P ratio of 1.67. For pure Mg, extrusion might retard corrosion and the release of Mg^2+^ because of grain refining, which led to less deposition of C, Ca, and P and a lower Ca/P ratio. The addition of Ag or extrusion at 7.1 for the Mg-Ag alloys might lead to increased release of Mg^2+^ and Ag^+^ by rapid galvanic degradation, which facilitated more C, Ca, and P deposition and a higher Ca/P ratio, whereas increased generation of H_2_ gas bubbles by rapid corrosion might disturb Ca and P deposition in some local positions, lowering the Ca/P ratio, as shown in Mg-6Ag-ER7.1. However, C deposition was not affected by the disturbance, which might have been caused by the high molecular weight of the organic compounds.

In addition, positions 4 and 13 were identified as some Mg-containing oxides, and position 6 was identified as particles formed by deposition. Positions 9, 11, and 15 were pits left by H_2_ bubbles. According to the EDS analysis, we conjectured that the bubbles hindered the deposition of organic and inorganic matter because the levels of C, Ca, and P on the pits were lower compared with those on other samples' surfaces.

**Figure 7:**
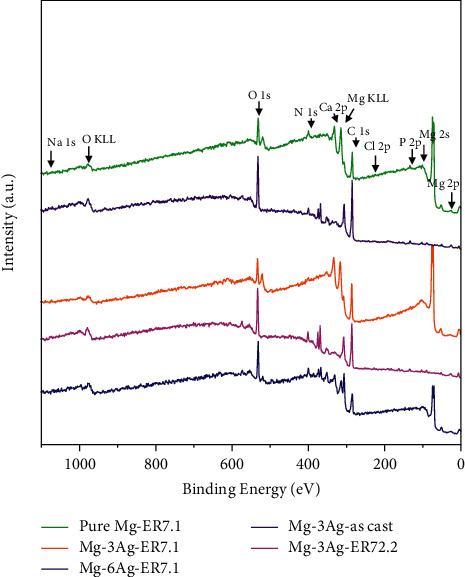
XPS results of the sample surfaces after an immersion test for 16 days.

To simplify the experiment and facilitate regulatory studies, five representative samples covering different Ag contents and extrusion ratios (pure Mg-ER7.1, Mg-3Ag-as cast/ER7.1/ER72.2, and Mg-6Ag-ER7.1) were selected for the following characterizations. The XPS results of these samples after the immersion test are shown in Figure 7. The compositions of the sample surfaces by XPS are shown in Table 5. The detected element signals were C 1s, N 1s, O 1s, Na 1s, P 2p, S 2p, Cl 2p, and Ca 2p, and the matrix elements were Mg 2s/2p and Ag 3d. As the Ag content increased, the C and Ca/P deposition increased. For the Mg-3Ag alloy, the ER7.1 alloy had the largest C and Ca/P deposition because of galvanic corrosion. The results also suggested that the test point was hardly influenced by H_2_ gas.

**Figure 8:**
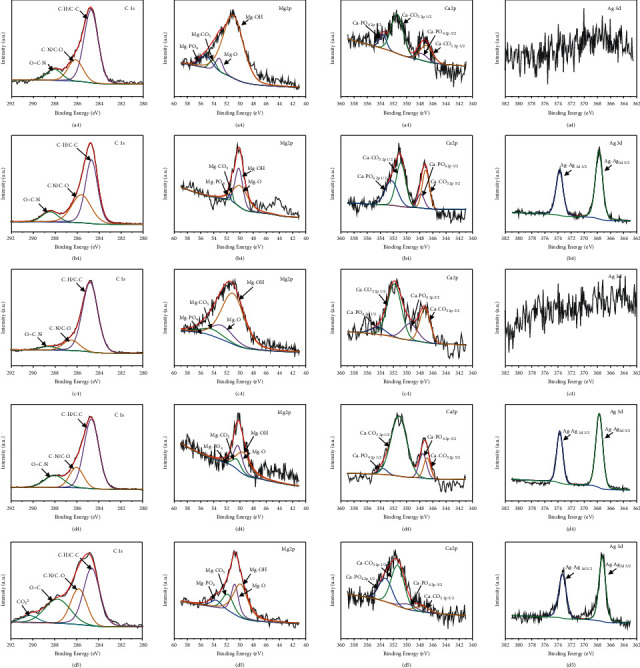
High-resolution spectra for C 1s, Mg 2p, Ca 2p, and Ag 3d signals of the surfaces of (a_1_–a_4_) pure Mg-ER7.1, (b_1_–b_4_) Mg-3Ag-as cast, (c_1_–c_4_) Mg-3Ag-ER7.1, (d_1_–d_4_) Mg-3Ag-ER72.2, and (e_1_–e_4_) Mg-6Ag-ER7.1 after an immersion test for 16 days.

The narrow scans of C 1s, Mg 2p, Ca 2p, and Ag 3d on the sample surfaces were fitted by MultiPak Software and are shown in Figure 8. The C 1s spectrum could be well fitted with four curves, which were identified as C-H/C-C (peak at 284.8 eV), C-N/C-O (peak at 285.4 eV-286.2 eV), O=C-N (peak at 288.3 eV), and CO_3_^2−^ (peak at 289.3 eV). The C-C/C-H signal might be mainly caused by atmospheric contamination, whereas the others might come from the amine or amide groups of organic matter, such as proteins. The Mg 2p spectrum could also be fitted by four curves whose peak signals were assigned to Mg-OH (49.3 eV), Mg-O (50.2 eV), Mg-CO_3_ (51.0 eV), and Mg-PO_4_ (51.6 eV) [23]. The Ca 2p spectrum was assigned to Ca-CO_3_ 2p_1/2, 3/2_ and Ca-PO_4_ 2p_1/2, 3/2_, and the Ag 3d spectrum could be identified as Ag-Ag 3d_3/2_ and 3d_5/2_ [32] which indicated the formation of Ag_2_O, according to the previous research [33].  Table 6 shows the atomic percentage of each chemical bond in their corresponding element for C 1s, Mg 2p, and Ca 2p. We found that in C 1s, the C-N/C-O peak and the O=C-N proportion generally increased as the Ag content increased. CO_3_^2−^ was only found on the Mg-6Ag sample surface. In Mg 2p, the main content was Mg-OH, and as the Ag content increased, more other bonds, such as Mg-O, Mg-CO_3_, and Mg-PO_4_, were detected. The amount of the detected content ranged (from high to low) as Mg-OH > Mg-O > Mg-CO_3_ > Mg-PO_4_. For Ca 2p for all samples, the main bond contents were Mg-CO_3_ > Mg-PO_4_. Moreover, we found that the addition of Ag led to more Mg-CO_3_ and Ca-PO_4_ bond formation, and this phenomenon might promote the activity of osteoblasts, which would modify the Ca/P ratio to resemble the bone mineral components [22].

**Figure 9:**
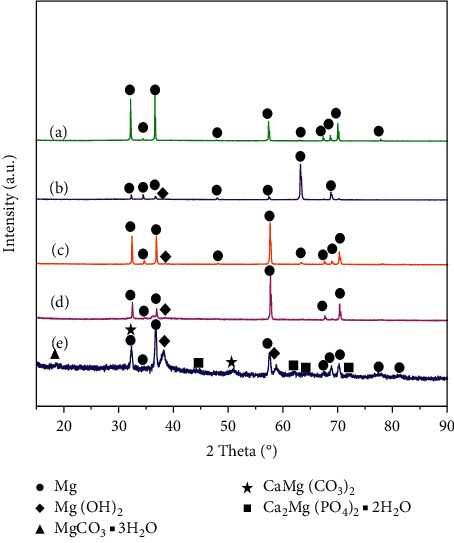
XRD results of the surfaces of (a) pure Mg-ER7.1, (b) Mg-3Ag-as cast, (c) Mg-3Ag-ER7.1, (d) Mg-3Ag-ER72.2, and (e) Mg-6Ag-ER7.1 after immersion test for 16 days.

Figure 9 presents the XRD results of the sample surfaces after 16 days of immersion. It should be noted that the XRD could only identify the crystals. The pure Mg sample surface was only detected with Mg peaks, whereas the Mg-3Ag (as cast, ER7.1, and ER72.2) sample surfaces showed Mg and Mg(OH)_2_ peaks. For the Mg-6Ag ER7.1 sample, besides Mg and Mg(OH)_2_, more peaks from MgCO_3_ 3H_2_O, CaMg(CO_3_)_2_, and Ca_2_Mg(PO_4_)_2_ 2H_2_O were detected. This showed that Ag might promote the crystallization process.

**Figure 10:**
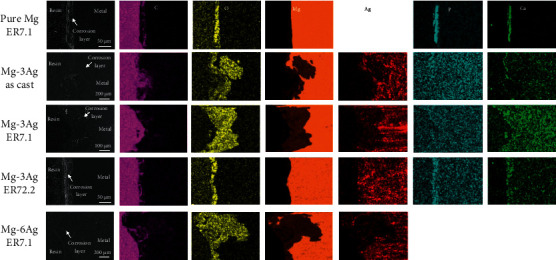
BSE images and chemical element mappings of the cross section of samples after an immersion test for 16 days.

Figure 10 shows the BSE images and chemical element mapping of the cress section of samples after the immersion test. According to the BSE images, the pure Mg-ER7.1 showed uniform corrosion with a product layer of∼40 *μ*m in thickness. As the Ag content increased, the corrosion accumulated and the corrosion morphology became heterogeneous with more local corrosion. For the Mg-3Ag alloys, an of ER7.1 accelerated local corrosion because more precipitate was present. Further extrusion (ER72.2) could modify the microstructure and refine the precipitates, which promoted uniform corrosion. Chemical element mapping indicated the deposition of C, O, P, and Ca elements on the sample surface. Oxygen was detected markedly, which indicated oxidization of the corrosion layers. Ca and P were detected, which demonstrated the promotion of calcium phosphate deposition by the Mg-Ag alloys. We also observed that Ca and P were hardly detected in the Mg-6Ag-ER7.1 sample, which indicated that rapid corrosion retarded calcium phosphate deposition on some local pits.

### 3.4. *In Vivo* Evaluation of Degradation Behavior

#### 3.4.1. Micro-CT

**Figure 11:**
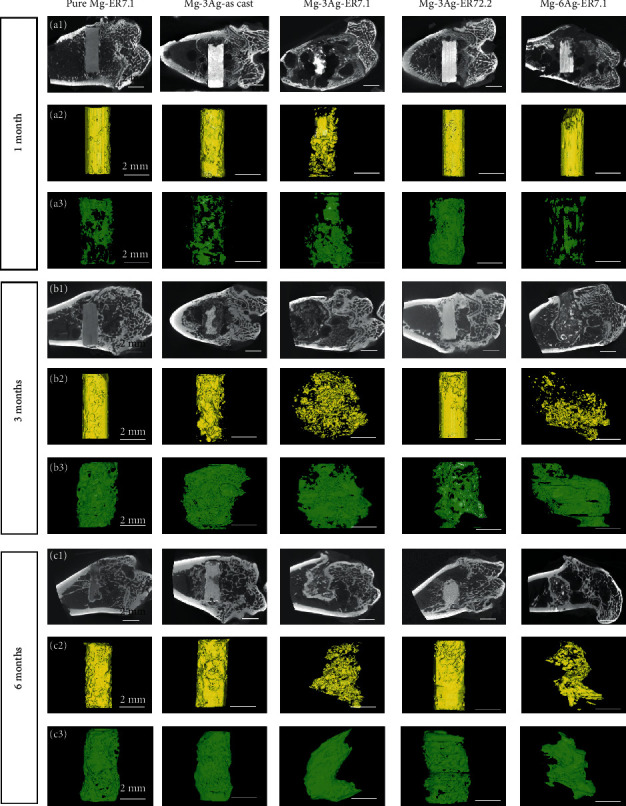
Micro-CT scans for (a_1_, b_1_, c_1_) two-dimensional slices and three-dimensional reconstructed images of (a_2_, b_2_, c_2_) implants and (a_3_, b_3_, c_3_) new bone after 1, 3, and 6 months of operation.

**Figure 12:**
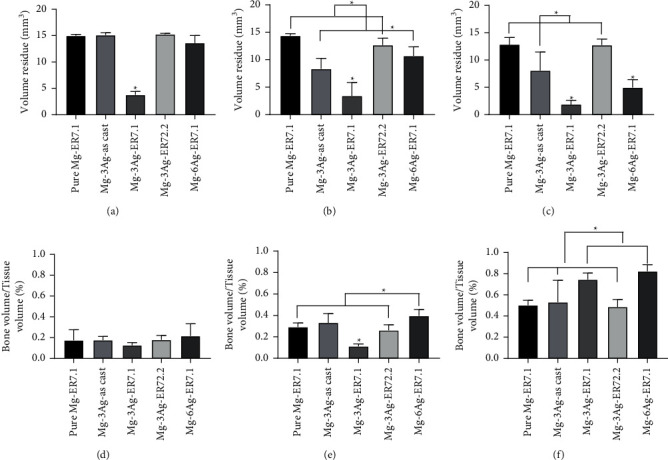
Implant volume residue after (a) 1, (b) 3, and (c) 6 months of operation. Bone volume/tissue volume (BV/TV) after (d) 1, (e) 3, and (f) 6 months of operation (^*∗*^*p* < 0.05).

The rat femoral condyle model was used to evaluate the performance of the Mg-Ag alloys *in vivo*.  Figure 11 shows the 2D radiographs and reconstructed micro-CT 3D images of the implants and new bone at 1, 3, and 6 months after surgery. To quantitatively determine the degradation behavior and the new bone growth, the volume remaining and the BV/TV were calculated and presented in Figure 12.

The pure Mg-ER7.1 implant degraded steadily with time, with few hydrogen gas bubbles appearing. The new bone volume increased as degradation proceeded, with no abnormal signs of osteolysis, deformity, or dislocation. For Mg-3Ag-as cast and Mg-3Ag-ER72.2, the implants degraded faster, with some gas bubbles appearing. The new bone volume was higher than that of pure Mg-ER7.1. For the Mg-3Ag-ER7.1 implant, degradation was very fast with many hydrogen gas bubbles appearing during the initial time after implantation, which might influence osteogenesis. After 1 month, the implant had already degraded into fragments, losing its original structure. The new bone volume was low at the early stages (1 and 3 months), but higher after 6 months of implantation. The Mg-6Ag-ER7.1 implant degraded slowly during the initial period (1 month), but increased after 1 month with some gas bubbles appearing. The implant also degraded into fragments at 3 months. The new bone generation was slow in the early stages (1 month) but became fast later.

**Figure 13:**
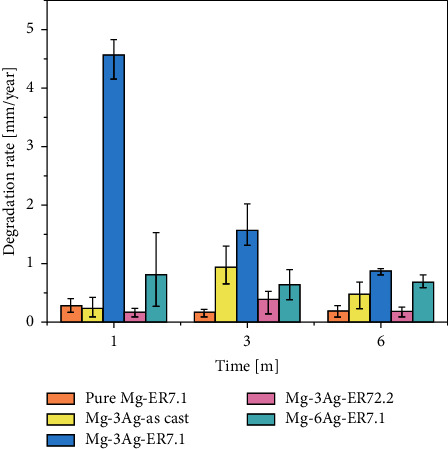
Degradation rate for pure Mg and Mg-Ag alloys *in vivo*.

The degradation rates *in vivo* were calculated based on the micro-CT data and are presented in Figure 13. The degradation rate fluctuated owing to competition, continuous galvanic corrosion, and thickening of the protective product layer. The effect of Ag addition and extrusion at 7.1 for the Ag-containing alloys might promote degradation, while the Mg-6Ag-ER7.1 alloy showed the reverse results. This might be because more Ag *in vivo* could retard degradation.

#### 3.4.2. Histological Evaluation

**Figure 14:**
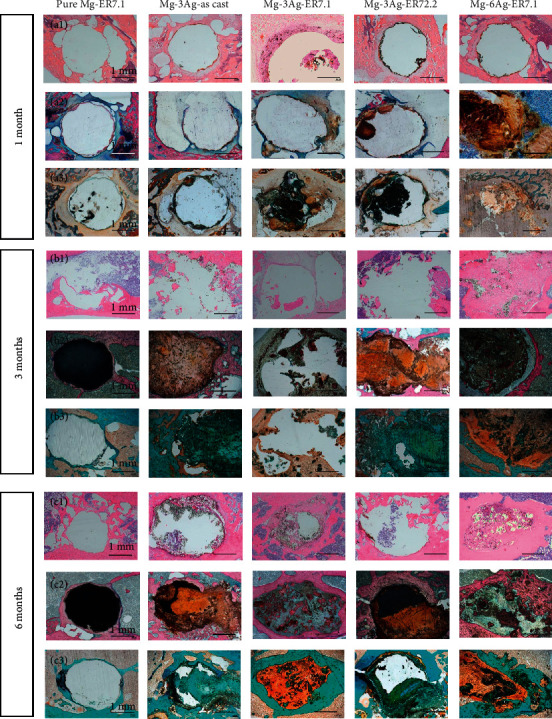
(a_1_, b_1_, c_1_) Hematoxylin-eosin (H&E), (a_2_, b_2_, c_2_) Methylene blue-acid fuchsin, and (a_3_, b_3_, c_3_) Goldner's trichrome stained images of the samples after 1, 3, and 6 months of operation.

Figure 14 shows the H&E, Goldner's trichrome, and Methylene blue-acid fuchsin staining of each group of materials. Upon H&E staining, the osteoblast nucleus appeared blue-black, the cytoplasm and the collagen fibers appeared pink, and the inflammatory cells appeared red.

For pure Mg-ER7.1, after implantation, there were few peripheral inflammatory cells, and collagen fibers that formed around the implant gradually thickened with prolonged implantation. Mg-3Ag-as cast and Mg-3Ag-ER72.2 showed similar results: there were fewer inflammatory cells around the implant and the collagen fibers formed around it were thicker than those around pure Mg-ER7.1 throughout the process. Furthermore, cartilage formed around 1 month after implantation was absorbed later. After 3 months, the internal cavity was relatively large, which represented the degradation product of the alloy corroded away during the tableting process. After 6 months, the void had decreased and the degradation products of the alloy were partially absorbed. For Mg-3Ag-ER7.1, at the early stages of implantation (1 and 3 months), a large cavity was formed, indicating that the material degraded rapidly. Osteoblasts gathered around it and gradually formed collagen fibers. In addition, inflammatory cells were observed. After 6 months, the degradation rate of the implant slowed down, and the collagen fibers gradually matured and thickened, with significantly decreased numbers of inflammatory cells. The results for Mg-6Ag-ER7.1 were similar to those for Mg-3Ag-ER7.1. The difference was that the degradation of the alloy slowed down and the surrounding collagen fibers thickened earlier (after 3 months of implantation).

Upon Goldner's staining, the original mineralized bone appeared green; the preliminary mineralized corrosion product appeared light green; the new bone appeared dark green; the osteoid and the osteoblasts appeared orange; and the inflammatory cells appeared deep orange. Upon methyl blue acid fuchsin staining, the original mineralized bone appeared pink; the preliminary mineralized corrosion product appeared orange; the new bone appeared purple-red; the osteoid appeared purple-gray; the osteoblasts appeared blue; and the inflammatory cells appeared dark blue. The pure Mg-ER7.1 material gradually degraded after implantation, and bubbles were produced around it, which increased after implantation for 1 and 3 months, but decreased after implantation for 6 months. On the whole, osteogenesis was good, and mature osteoblasts existed in the new bone. Mg-3Ag-as cast and Mg-3Ag-ER72.2 showed similar results: at 1 month after implantation, the material slowly degraded with a ring of immature new bone and bubbles; at 3 months after implantation, the cavity was filled with primarily mineralized degradation products, and there were osteoid and a small amount of normal nascent bone at the interface with the mineralized bone. After 6 months of implantation, the degradation products of initial mineralization were reduced and the amount of new bone around the implant increased. Mg-3Ag-ER7.1 was hollow and large in the preimplantation periods (1 and 3 months), indicating that the material degraded rapidly and produced many degradation products inside, with a part of it being preliminarily mineralized and many osteoid tissues at the same time. After 6 months of implantation, osteoblasts and deeply stained inflammatory cells were observed, and the cavity was filled with osteoid and surrounded by new bone. For Mg-6Ag-ER7.1, after 1 month of implantation, the cavity was already filled with osteoid and inflammatory cells. With the prolongation of implantation time, the cavity was gradually reduced while the osteoid and the inflammatory tissue were gradually converted into new bone.

## 4. Discussion

### 4.1. The Degradation Rates of Mg-Ag Alloys *In Vitro* and *In Vivo*

We first determined the composition of the alloy, which showed that the contents of the impurities Cu, Fe, and Ni were all low and did not influence the alloy corrosion. The initial microstructure of the alloy, such as the proportion of precipitates and the grain sizes of the samples, has a significant effect on the alloy degradation rates [34, 35].

The Mg bulk alloy and the precipitate could form a microgalvanic cell because of the difference in potential [36, 37]. The Mg matrix acted as the anode and the precipitates, Mg_54_Ag_17_ and Mg_4_Ag in this study, acted as the cathodes. This increased the degradation rate. The effect of grain size on degradation was not straightforward and depended on the materials and the environment [34]. In a mild environment that was favorable for the formation of a protective product layer, a fine grain might promote a more dense and tough layer because it might relieve the stresses in the layer caused by the vacancy provided through the grain boundary [38–40]. However, when the levels of aggressive ions (such as Cl^−^) were high, the product layer might lose its protective effect, and a fine grain might increase the degradation rate because more of the grain boundary is exposed [39, 41]. Our *in vitro* and *in vivo* studies both belonged to the mild environment situation.

In addition, the microstructure defects, such as dislocations and twins, could result in residual stress in the alloy, thus leading to an increase in the degradation rate. Hot extrusion can have a good recovery effect, which eliminates defects when parameters such as the extrusion temperature and speed are set appropriately.

Generally, at the beginning of *in vivo* and *in vitro* tests, Mg corroded to produce Mg^2+^, H_2_, and OH^−^ following the reaction:

(3) $$\begin{array}{c} \text{Mg}+{2\text{H}}_{2}\text{O}⟶{\text{Mg}}^{2+}+{2\text{OH}}^{-}+\text{H}2↑ \end{array}$$

Later, Mg^2+^ might react with OH^−^ to produce magnesium hydroxide on the substrate surface following the reaction:

(4) $$\begin{array}{c} \text{Mg}+{2\text{OH}}^{-}⟶\text{Mg}{\left(\text{OH}\right)}_{2} \end{array}$$

The degradation rate of the Mg-Ag alloys was higher than that of pure Mg because of intense galvanic corrosion. Extrusion could refine the grain, which mainly decreased the degradation rate in the early stages. After immersion for a period of time, the degradation rate of the Mg-Ag alloys decreased more than that of pure Mg, resulting from the formation of a protective compact product layer [42, 43]. Then, corrosive ions, such as Cl^−^, might penetrate the substrate, causing pits on the surface. The gas bubbles generated by degradation might also lead to cracks in the product layer. These processes progressively consumed the Mg matrix and accumulated the degradation product layer, resulting in continuous degradation [28]. This caused a fluctuation of the degradation rate during the immersion test. For pure Mg, the main effect on degradation is the grain size, and extrusion may refine the grain, which decreased the degradation rate. For the Mg-Ag alloys, the addition of Ag and a lower extrusion ratio (7.1) led to more precipitates in the Mg matrix, which promoted rapid galvanic corrosion. A higher extrusion ratio (72.2) might lead to the precipitates dissolving in the Mg matrix, although the grain size increased a little. This resulted in a lower degradation rate at the later stages of the immersion test.

The degradation rates of the Mg-Ag alloys *in vivo* were generally lower than those *in vitro*, which reflected the fact that the material implanted *in vivo* directly contacts bone tissue, and the degradation environment is milder than that *in vitro*. Another reason might be that the *in vivo* observation time was relatively long, resulting in a stable degradation layer forming on the surface of the material, inhibiting further degradation [44].

Notably, the degradation rate of Mg-6Ag-ER7.1 was higher than that of Mg-3Ag-ER7.1 *in vitro*, whereas the reverse was true in *in vivo*. The reasons might be that in *in vivo*: (1) more precipitates formed after the addition of a large amount of Ag, which can form a network protection effect to delay corrosion, similar to that observed in the AZ series [45]. (2) Ag is not only dissolved in the precipitate but also exists in the matrix, which could make the alloy surface easier to oxidize, forming a protective film and retarding corrosion. (3) The addition of more Ag could reduce the potential difference between the precipitate and the matrix, thus slowing the electrochemical reaction [46]. Taken together, the degradation behavior was complicated and might be affected by many factors.

### 4.2. The Degradation Product of Mg-Ag Alloys *In Vitro*

Mg^2+^ and Ag^+^ can promote the deposition of C-containing organic matter and calcium phosphate on sample surfaces. Based on the results of EDS and XPS analysis, for pure Mg, the grain size could be refined by extrusion, which decreased the corrosion rate and the release of Mg^2+^. This slowed down the deposition of C-containing organic matter and calcium phosphate and decreased the Ca/P ratios. For the Mg-Ag alloys, the addition of Ag and small extrusion ratio extrusion (ER7.1) promoted the rapid galvanic corrosion of the alloy, which increased the release of Mg^2+^ and Ag^+^ and accelerated the deposition of C-containing organic matter and calcium phosphate. However, at the same time, rapid corrosion also produced many H_2_ bubbles, which inhibited the deposition of calcium phosphate in some areas; however, the deposition of C-containing organic matter is less affected by this interference because of its high molecular weight. The narrow scan (Figure 8) and the proportion of each product formed on the surface (Table 6) were analyzed by Multi-Pak Software. We found that the main bonds in the surface were C-C/C-H in the C 1s group, Mg-OH in the Mg 2s group, and Ca-CO3 in the Ca 2p group, which identified the product composition. As the content of Ag increased, more Mg-CO_3_ and Ca-PO_4_ bonds were formed, which might promote osteogenesis. Ag could also promote the crystallization of more products deposited on the surface, as shown in Figure 9.

Marco et al. [47] reported that the immersion of the Mg-2Ag alloy in DMEM resulted in a two-layer product structure, while in our study, we did not observe an obvious two-layer structure. This might be because we added FBS, which disturbed the deposition reaction and made the two-layer boundary blurred. Another reason might be a characterization issue: a higher resolution and a larger characterization size might be needed to solve this problem.

### 4.3. The Histological Response of the Mg-Ag Alloys *In Vivo*

In the *in vivo* implantation experiment, pure Mg had few degradation products and resulted in good osteogenesis, and no inflammatory reaction was observed. In the Mg-Ag group, the degradation rate affected the formation of degradation products, the osteogenic process, and the inflammatory reaction: (1) for alloys with slightly slower degradation (Mg-3Ag-as cast and Mg-3Ag-ER72.2), degradation products with initial mineralization and a small amount of osteoid substance appeared at the implantation site. With the increase in the degradation rate, more degradation products for initial mineralization were generated. No obvious inflammatory reaction was observed around the implantation site. (2) For the alloys with rapid degradation (Mg-3Ag-ER7.1 and Mg-6Ag-ER7.1), many osteoid and inflammatory fibers appeared at the implantation site. The higher the degradation rate, the more osteoid and inflammatory fibers appeared in the implant site.

Mg alloy degradation releases Mg^2+^, which plays a key role in the binding interaction of the cell surface integrin family with its ligand protein [48]. Mg^2+^ can also promote cell adhesion and proliferation of bone marrow mesenchymal stem cells on the implant surface [49, 50]. The first step of osteogenesis is the deposition of hydroxyapatite, and the microalkaline environment generated by the degradation of magnesium alloy promotes the deposition of Ca and P, which is the beginning of the formation of hydroxyapatite and provides good conditions for subsequent bone repair [51].

Degradation of the Mg-Ag alloys also releases Ag^+^, which affects the behavior of osteoblasts and osteoclasts. According to previous reports, after being implanted *in vivo*, Mg-2Ag promotes osteoblast activity but inhibits osteoclast activity, which is related to the effect of Ag^+^. Ag^+^ results in a large amount of callus around the implant, which will gradually transform into mature new bone over time [24]. Ag^+^ can also bind strongly to mercaptans, metallothionein, albumin, and macroglobulin *in vivo*, resulting in complex formation [52, 53]. Its antibacterial property is related to the Ag^+^ concentration at the infected site. The Ag concentration is determined by the amount of Ag in the Mg-Ag alloy and its release rate. It is preferable to alloy as much silver as possible with pure Mg to ensure effective antibacterial properties, based on a controlled degradation rate [54]. It is also important to note that the total amount of Ag in an Mg-Ag alloy should not exceed the amount that can cause silver poisoning in humans.

In this paper, the alloys with a lower degradation rate produced a preliminary mineralized degradation product, which might be the early stage of callus formation, according to previous reports. However, when the alloys degraded too quickly, H_2_ bubbles accumulated, which led to the appearance of inflammation. The antibacterial effect of Ag^+^ might be weaker than the inflammatory response generated by the bubbles. However, in the later period (6 months), osteogenesis outside the inflammatory tissue was still very good, which is worthy of further study.

## 5. Conclusion

In this paper, the Ag content and the hot extrusion with different ERs were studied to clarify their effects on the degradation behavior of Mg-Ag alloys *in vitro* and *in vivo*. Some conclusions can be drawn as follows:

1. Ag could either accelerate degradation by galvanic corrosion or hinder degradation by forming a protective oxide network especially *in vivo*. Ag could also promote the crystallization of products, biomineralization, and the deposition of organic matter.
2. Extrusion could refine the grain size and alter the proportion of the precipitates. For pure Mg, extrusion decreased the degradation rate because of grain refinement. For the Mg-Ag alloys, extrusion at a lower ratio (7.1) resulted in more serious degradation the higher ratio (72.2) because of the proportion of precipitates.
3. Rapid degradation led to a large release of Mg^2+^ and Ag^+^, which resulted in increased deposition of organic matter and calcium phosphate. However, the H_2_ bubbles caused by serious degradation might inhibit product deposition in some local points.
4. *In vivo*, the alloys with rapid degradation (such as Mg-3Ag-ER7.1 and Mg-6Ag-ER7.1) induced a strong inflammatory response, and osteogenesis occurred around the inflammatory tissue. The alloy with slower and more appropriate degradation (such as Mg-3Ag-as cast and Mg-3Ag-ER72.2) might lead to callus formation and favorable osteogenic properties.

## Figures and Tables

**Table 1:** Mass composition and average density of pure Mg and Mg-Ag alloys.

Materials	Ag (wt.%)	Cu (wt.%)	Fe (wt.%)	Ni (wt.%)	Density (g/cm^3^)
Pure Mg	<0.001	<0.001	0.0022	<0.001	1.74
Mg-3Ag	2.78	<0.001	0.0019	<0.001	1.77
Mg-6Ag	6.34	<0.001	<0.001	<0.001	1.84

**Table 2:** Average proportion of precipitates and grain sizes of samples.

Sample	Average proportion of precipitates	Grain size (*μ*m)	
		Average	Standard deviation
Pure Mg-as cast	—	489.99	24.11
Pure Mg-ER7.1	—	77.25	16.80
Pure Mg-ER72.2	—	40.35	11.77
Mg-3Ag-as cast	0.267	288.24	25.91
Mg-3Ag-ER7.1	0.298	16.81	5.60
Mg-3Ag-ER72.2	0.253	37.72	14.66
Mg-6Ag-as cast	0.302	87.76	8.19
Mg-6Ag-ER7.1	0.377	23.83	8.64
Mg-6Ag-ER72.2	0.303	28.44	11.12

**Table 3:** Electrochemical parameters of pure Mg and Mg-Ag alloys after immersion in DMEM + 10% FBS for 1 day.

Samples	*E*_corr_ (V/SCE)	*I*_corr_ (A cm^−2^)
Pure Mg-as cast	−1.57	1.42E-05
Pure Mg-ER7.1	−1.57	4.40E-06
Pure Mg-ER72.2	−1.52	4.15E-06
Mg-3Ag-as cast	−1.44	4.65E-05
Mg-3Ag-ER7.1	−1.36	1.11E-05
Mg-3Ag-ER72.2	−1.50	1.04E-05
Mg-6Ag-as cast	−1.41	4.78E-05
Mg-6Ag-ER7.1	−1.51	1.44E-05
Mg-6Ag-ER72.2	−1.46	8.47E-06

**Table 4:** Composition of the sample surfaces by EDS after an immersion test for 16 days.

Samples	Position	Elements/at.%												
		C	N	O	Na	Mg	Si	P	S	Cl	K	Ca	Ag	Ca/P
Pure Mg-as cast	1	10.98		57.68	0.81	8.81		11.40				10.32		0.91
	2	12.59		52.74	0.71	9.74	0.44	11.98		0.45		11.35		0.95
Pure Mg-ER7.1	3	12.45		53.12	1.03	7.66		13.11		0.32		12.31		0.94
	4	11.77		56.78	2.43	16.04		6.12		0.35		6.50		1.06
Pure Mg-ER72.2	5	12.87		54.57	0.84	15.27	1.11	9.57		0.09		5.68		0.59
	6	14.32		50.71	0.76	13.58	0.50	10.90				9.23		0.85
Mg-3Ag-as cast	7	16.76		48.11	1.31	17.02		6.62		0.46		7.15	2.56	1.08
	8	19.35		58.15	1.68	7.33		6.19		0.72		6.48	0.10	1.05
	9	18.25		52.06	1.30	17.53		5.24		0.47		5.09	0.06	0.97
Mg-3Ag-ER7.1	10	18.53		52.96	1.82	16.30		4.90		0.59		4.85	0.05	0.99
	11	20.91		51.23	1.77	17.96		3.57	0.62	0.84		2.60	0.49	0.73
Mg-3Ag-ER72.2	12	18.74		46.46	4.11	18.53	0.40	4.96		2.32		3.92	0.56	0.79
	13	19.19		55.56	2.04	16.74		2.93				2.23	1.04	0.76
Mg-6Ag-as cast	14	17.76		50.56	1.03	6.52		7.73		0.66		15.22	0.52	1.97
	15	16.18		47.89	1.10	9.89		10.31	0.46	0.50		13.19	0.49	1.28
Mg-6Ag-ER7.1	16	22.57		52.54	1.75	21.70				1.00			0.44	
	17	23.42	7.13	45.72		22.43			0.30				0.99	
Mg-6Ag-ER72.2	18	16.17		53.21	1.12	12.37		8.42				8.70		1.03
	19	16.45		50.80	0.99	9.22		5.01			0.35	9.97	0.25	1.99
	20	12.54		50.46	1.04	15.60		9.35				10.95	0.06	1.17

**Table 5:** Composition of the sample surfaces by XPS after an immersion test for 16 days.

Samples	Atomic concentration/at.%										
	C 1s	N 1s	O 1s	Na 1s	Mg 2s	P 2p	S 2p	Cl 2p	Ca 2p	Ag 3d	Ca 2p/P 2p
Pure Mg-ER7.1	53.05	10.02	7.20	0.00	26.38	1.59	0.00	0.00	1.49	0.28	0.94
Mg-3Ag-as cast	60.45	4.62	25.76	1.23	2.54	1.81	1.13	0.16	1.32	0.98	0.73
Mg-3Ag-ER7.1	61.36	3.11	6.84	0.39	23.85	1.42	0.69	0.00	1.93	0.40	1.36
Mg-3Ag-ER72.2	58.34	4.34	27.16	0.26	4.88	1.48	0.30	0.00	1.50	1.74	1.01
Mg-6Ag-ER7.1	47.16	8.56	20.70	0.00	17.05	1.37	0.00	1.17	2.73	1.26	1.99

**Table 6:** Composition of the sample surfaces by high resolution spectra XPS after an immersion test for 16 days.

Samples	C 1s (100 at.%)				Mg 2p (100 at.%)				Ca 2p (100 at.%)	
	CO_3_^2−^	O=C-N	C-N/C-O	C-C/C-H	Mg-PO_4_	Mg-CO_3_	Mg-O	Mg-OH	Ca-PO_4_	Ca-CO_3_
Pure Mg-ER7.1	0.00	11.91	19.49	68.60	2.07	7.38	0.59	89.96	21.05	78.95
Mg-3Ag-as cast	0.00	11.41	18.38	70.22	2.10	2.85	43.19	51.86	62.92	37.08
Mg-3Ag-ER7.1	0.00	3.99	13.16	82.85	0.96	9.92	19.39	69.73	25.64	74.36
Mg-3Ag-ER72.2	0.00	14.98	18.15	66.87	0.00	13.39	33.60	53.01	17.78	82.22
Mg-6Ag-ER7.1	7.39	24.41	23.11	45.10	9.31	18.50	22.91	49.29	32.22	67.78

## Data Availability

The data are available on request from the corresponding authors.
